# Nitroxoline suppresses metastasis in bladder cancer via EGR1/circNDRG1/miR-520h/smad7/EMT signaling pathway

**DOI:** 10.7150/ijbs.69373

**Published:** 2022-08-08

**Authors:** Liangliang Ren, Minxiao Jiang, Dingwei Xue, Huan Wang, Zeyi Lu, Lifeng Ding, Haiyun Xie, Ruyue Wang, Wenqin Luo, Li Xu, Mingchao Wang, Shicheng Yu, Sheng Cheng, Liqun Xia, Haifeng Yu, Peng Huang, Naijin Xu, Gonghui Li

**Affiliations:** 1 Department of Urology, Sir Run Run Shaw Hospital, Zhejiang University School of Medicine, Hangzhou, China.; 2 Department of Urology, Okayama University Graduate School of Medicine, Dentistry and Pharmaceutical Sciences, Okayama, Japan.; 3 Department of Urology, Zhujiang Hospital, Southern Medical University, Guangzhou, China.

**Keywords:** Bladder cancer, nitroxoline, metastasis circNDRG1, microRNA

## Abstract

Bladder cancer is one of the most common and deadly cancer worldwide. Current chemotherapy has shown limited efficacy in improving outcomes for patients. Nitroxoline, an old and widely used oral antibiotic, which was known to treat for urinary tract infection for decades. Recent studies suggested that nitroxoline suppressed the tumor progression and metastasis, especially in bladder cancer. However, the underlying mechanism for anti-tumor activity of nitroxoline remains unclear.

**Methods:** CircRNA microarray was used to explore the nitroxoline-mediated circRNA expression profile of bladder cancer lines. Transwell and wound-healing assay were applied to evaluate the capacity of metastasis. ChIP assay was chosen to prove the binding of promotor and transcription factor. RNA-pulldown assay was performed to explore the sponge of circRNA and microRNA.

**Results:** We first identified the circNDRG1 (has_circ_0085656) as a novel candidate circRNA. Transwell and wound-healing assay demonstrated that circNDRG1 inhibited the metastasis of bladder cancer. ChIP assay showed that circNDRG1 was regulated by the transcription factor EGR1 by binding the promotor of host gene NDRG1. RNA-pulldown assay proved that circNDRG1 sponged miR-520h leading to the overexpression of smad7, which was a negative regulatory protein of EMT.

**Conclusions:** Our research revealed that nitroxoline may suppress metastasis in bladder cancer via EGR1/circNDRG1/miR-520h/smad7/EMT signaling pathway.

## Introduction

Bladder cancer is one of the most common cancer around the world, with an estimated 81,400 new cases and 17,980 new deaths in United States in 2020 1. Bladder cancer is staged by the measure of depth of bladder wall invasion, including non-muscle-invasive bladder cancer (NMIBC) and muscle-invasive bladder cancer (MIBC) 2, 3. NMIBC accounts for about 80% of all newly cases. Bacille Calmette-Guérin (BCG) is the recommended treatment after surgery 4. However, some patients may not benefit from the treatment of BCG for drug resistance or unbearable side-effect 5. Therefore, it is urgent and important to explore new drug candidates for the treatment of bladder cancer.

Nitroxoline is an antibiotic and known to treat for urinary tract infection 6. Recently, several studies have reported that nitroxoline may be a potential antineoplastic drug in bladder cancer, myeloma, glioma, pancreatic cancer, prostate cancer and so on 7-11. Our recent research suggested that nitroxoline, as a novel STAT3 inhibitor, could induce P-gp reversal, G0/G1 arrest, and apoptosis in drug-resistant urothelial bladder cancer 12. Notably, completed multicenter phase II clinical trials have demonstrated the efficacy and safety of nitroxolines in chemotherapy/BCG instillation unresponsive high-risk NMIBC patients, with significantly better clinical efficacy than the currently clinically used bladder instillation chemotherapeutic agents (e.g., mitomycin) and immunologic agents (e.g., BCG). The clinical use of nitroxoline has advantages compared with chemotherapeutic instillation. First, its efficacy is superior to that of conventional chemotherapeutic instillation in patients with recurrent chemotherapy by perfusion, allowing some high-risk patients to avoid total cystectomy. Second, nitroxoline is taken orally instead of by irrigation of bladder, which is easier to be accepted by patients. Third, nitroxoline displays a superior human safety with no serious side effects. However, the specific mechanism underlying nitroxoline inhibits the progression of bladder cancer is still not clear. Our previous research has found that nitroxoline could reverse Epithelial-Mesenchymal Transition (EMT) and enhance anti-tumor immunity in bladder cancer 13. But the upstream mechanism remains unknow and needs further researches. Therefore, in this study, we try to interpret nitroxoline-mediated anti-tumor effect from non-coding RNAs perspective.

CircRNAs are a class of endogenous noncoding RNAs with a special circular structure that lacks 5' end caps and 3' end poly(A) tails 14. CircRNAs are more stable and longer transcription half-life due to the unique circular structure. And circRNAs usually play a biological role via sponging microRNA or binding protein 15, 16. Many researches have confirmed that circRNAs contributed to bladder cancer, including proliferation, metastasis, apoptosis and so on 17, 18. But few researches report the role and mechanism of circRNAs in antitumor drugs-mediated inhibition of bladder cancer progression.

In this study, we first verified that nitroxoline suppressed the metastasis of bladder cancer in vivo and vitro. CircRNA microarrays were used to analyze the expression of circRNAs between nitroxoline-treated and untreated group. The level of circNDRG1 was markedly upregulated and circNDRG1 suppressed the metastasis of bladder cancer. Then we found that transcription factor EGR1 regulated the level of circNDRG1 positively by binding the promotor domain of NDRG1. Further researches revealed that circNDRG1 sponged miR-520h contributing to the translation of smad7, which was a negative regulatory protein of EMT. In summary, nitroxoline suppressed metastasis in bladder cancer via the signaling pathway of EGR1/circNDRG1/miR-520h/smad7/EMT. Nitroxoline may be a potential candidate and our study may provide a new idea and theoretical basis for the treatment of bladder cancer.

## Materials and Methods

### CircRNA microarray and reagent

T24, UM-UC-3, TCCSUP and J82 cell lines were treated by nitroxoline (20 µM) for 48 hours. The different expression of circRNA between nitroxoline-treated and untreated group was analyzed by circRNA microarray (Lianchuan, China). There were 88,701 circRNAs to be tested. Nitroxoline was purchased from Selleck (catalog: S4591).

### Cell lines and culture

Human bladder cancer cell lines (T24, UM-UC-3, TCCSUP, J82) were purchased from the Cell Bank of the Chinese Academy of Science (Shanghai, China). Cells were cultured in the medium with 10% fetal bovine serum (Gibco, Australia). T24 cell was maintained in RPMI-1640 medium (Gibco, China) and other cells were cultured in MEM medium (Gibco, China). The cell incubator was set at 37 °C with 5% CO_2_ in a humidified atmosphere.

### Transwell and wound-healing assay

Transwell inserts (Corning, USA) with a pore size of 8 µm were used to evaluate the cell migration ability. Cells were seeded into the transwell inserts with serum-free medium while the bottom of inserts with 10% serum medium. After 24 hours, 4% paraformaldehyde was used to fix the cells and 0.3% crystal violet was applied to stain the moved cells.

For wound-healing assay, cells were cultured in 6-well plates to 100% confluence. 200µL pipette tip was used to scratch the cell monolayer. Then, the scratched cells were cultured in serum-free medium for 24 hours. An Olympus BX51 upright microscope was adopted to obtain the images. Migration rate = (wound area (0h) - wound area (24h)) / wound area (0h). All assays were repeated 3 times.

### Quantitative real-time PCR (RT-qPCR) and RT-PCR

The total RNA from cells were isolated using TRIzol reagent (CWBIO, China). RNA was reverse transcribed using PrimeScript RT Master Mix (CWBIO, China). SYBR Green (CWBIO, China) was used in RT-qPCR assay and the relative quantification of RNA were calculated by 2^-ΔΔCT^ method based on the internal reference of β-actin, GAPDH or U6. The whole primer sequences were listed in Supplementary (Table S1).

### Cell transfection

All the si-RNAs (RIBBIO, China) were transfected into the cells using RFect reagent (BAIDAI, China). The sequences of si-RNAs were listed in Supplementary (Table S1). All the overexpression plasmid (GENE, China) were transfected into the cells using Lipofectamine 3000 (Invitrogen, USA). The overexpression plasmid of circNDRG1 GV486 vector and GV658 vector were purchased from Shanghai Gene Co., Ltd.

### Western-blotting assay

The cell protein lysates were mixed with RIPA lysis buffer supplemented with PMSF and protein loading buffer. Then, the lysates were separated by SDS-polyacrylamide gel electrophoresis and transferred to PVDF membrane. The following antibodies, monoclonal anti-GAPDH (Catalogue #5174, 1:1000), monoclonal anti-E-cadherin (Catalogue #3195, 1:1000), monoclonal anti-N-cadherin (Catalogue#13116, 1:1000), monoclonal smad2/3 (Catalogue#8685T, 1:1000) were purchased from Cell signaling Technology; monoclonal anti-EGR1 (Catalogue#55160, 1:1000) was purchased from abcam; monoclonal anti-samd7 (Catalogue#66478-1-lg, 1:1000) was purchased from proteintech; monoclonal smad2/3-phosphorylation (Catalogue#abs130992, 1:1000) were purchased from Absin; anti-mouse and anti-rabbit IgG secondary antibody were purchased from Cell signaling Technology.

### Chromatin immunoprecipitation (ChIP) assay

ChIP assay kit (Cell signaling Technology, USA) was used to identify the relationship between protein and DNA-sequence as described in manufacturer's instructions. After cell lysis, isolated nuclei were subjected to sonication for chromatin fragmentation. Briefly, cells were crosslinked with 1% formaldehyde. Sheared chromatin was diluted in diluted buffer and divided into aliquots for immunoprecipitation. Antibody-chromatin complexes were captured using magnetic protein A/G beads. The primer of NDRG1 promotor was listed in supplementary (Table S1).

### RNA-pulldown assay

Biotin-labelled circNDRG1 probe was used to bind circNDRG1 through streptavidin agarose and microRNAs were sponged by circNDRG1. The total RNA of complex was extracted and RT-qPCR assay was used to analyze the difference. The sequence of Biotin circNDRG1 was listed in Supplementary (Table S1).

### RNA immunoprecipitation (RIP) assay

MagnaRIP RNA-Binding Protein Immunoprecipitation Kit (Millipore, USA) was used to identify the binding of protein and RNA. All steps were performed following the manufacturer recommendations. The monoclonal anti-AGO2 (Catalogue#186733, 1:50) was purchased from abcam.

### Dual luciferase reporter assay

The plasmids of luciferase (GENE, China) were constructed by GV272 vector. The Firefly and Renilla luciferase activity were quantified by Dual-Luciferase Report Kit (Promega, USA).

### Lentiviral transduction and tumor xenograft experiments

Lentiviruses expressing short hairpin RNA (shRNA) targeting circNDRG1 and lentivirus expressing negative control shRNA (NC) were purchased from GenePharma (Shanghai, China).

BALB/c nude mice were obtained from the Laboratory Animal Center of Sir Run Run Shaw Hospital (Hangzhou, China) and the whole experiments were approved by the Institutional Ethics Committee of Sir Run Run Shaw Hospital. Luciferase-labelled UM-UC-3 cells were injected into Five-week-old female nude mice by tail veins (2×10^6^ resuspended in 200μl PBS). Nitroxoline (30mg/kg per mouse, fed for 2 days and rest for 1 day) by gavage was fed for 8 weeks in NC/circNDRG1-sh groups 13. In Vivo Image System (IVIS) was used to evaluate the tumor growth.

### Statistical analysis

Statistical analysis was performed by GraphPad Prism 8. Student T-test was used to test the statistical significance of group differences (* *P*<0.05, ** *P*<0.01, *** *P*<0.001). Date was showed by mean ± standard-deviation (SD).

## Results

### Nitroxoline suppresses the metastasis of bladder cancer in vitro and vivo

**Figure 1:**
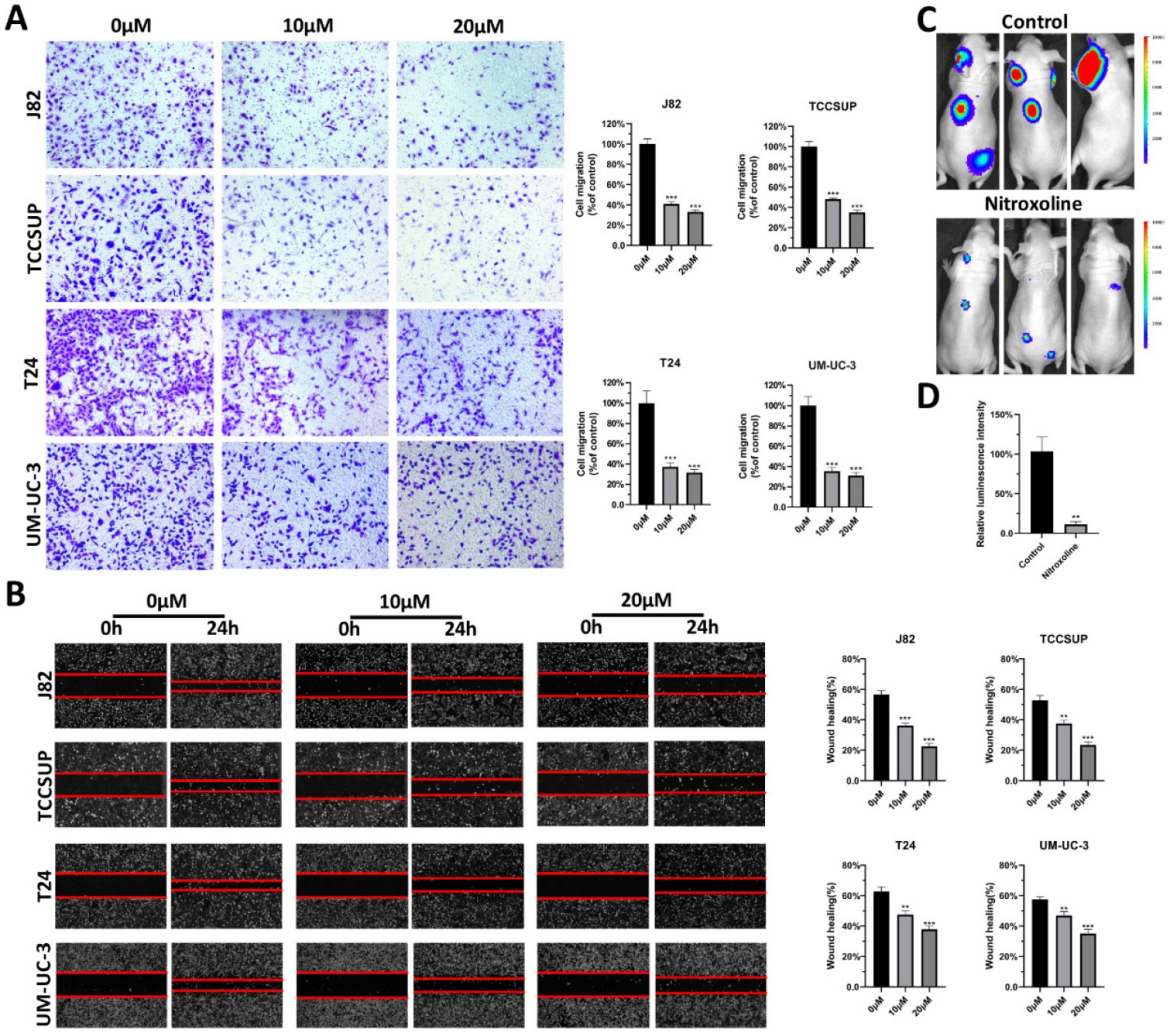
** Nitroxoline suppressed the metastasis of bladder cancer in vitro and in vivo. (A and B)** Transwell assay and wound-healing assay were performed to evaluate migration of bladder cancer cells. **(C)** Luciferase-labelled UM-UC-3 cells were injected into nude mice by tail veins for experimental metastasis. **(D)** Bioluminescent signals were measured by the IVIS imaging system. *P<0.05, **P<0.01 and ***P<0.001.

To verify the effect of nitroxoline on metastasis, transwell assay was performed on bladder cancer cell lines, including J82, TCCSUP, T24 and UM-UC-3 cells. The results indicated that nitroxoline decreased the migration capability of bladder cancer cells (Fig. 1A). And the similar results were observed in wound-healing assay. Bladder cancer cells treated with nitroxoline (10 and 20 µM) healed the wound more slowly than untreated cells (Fig. 1B). Furthermore, we established a tail vein injection model with nude mice to investigate the effects of nitroxoline on metastasis in vivo. The relative luminescence intensity in nitroxoline treated group was weaker than in control group, indicating nitroxoline relieved the metastasis of bladder cancer validly (Fig. 1C-D).

### CircNDRG1 is up-regulated after nitroxoline treatment

**Figure 2:**
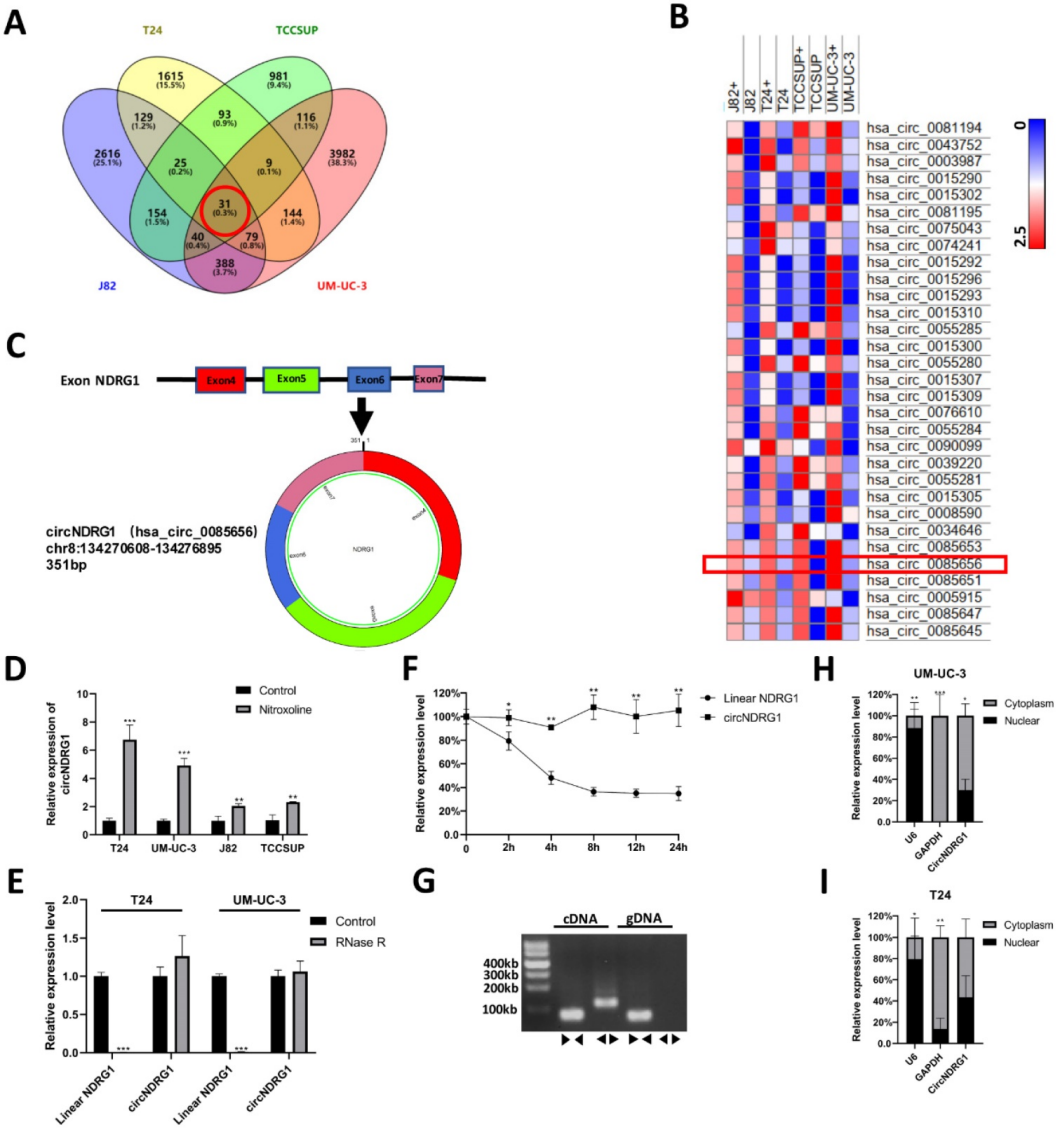
** CircNDRG1 was up-regulated after nitroxoline treatment. (A)** Venn diagram of the intersection among up-regulated circRNAs in 4 bladder cancer cells. **(B)** Heatmap of circRNA-seq analysis showed top 31 differentially high expressed circRNAs of 4 bladder cancer cells. **(C)** Schematic illustration demonstrates circNDRG1 formation via exons 4-7 circularization of NDRG1. **(D)** RT-qPCR was utilized to analyze the circNDRG1 expression. **(E)** RT-qPCR was used to determine the abundances of circNDRG1 and linear mRNA NDRG1 in T24 and UM-UC-3 cells treated with RNase R. **(F)** Transcription half-life of circNDRG1 and linear mRNA NDRG1 was demonstrated at the indicated times after adding actinomycin D. **(G)** Southern-blotting was used to analyze the amplification of divergent (◀▶) and convergent (▶◀) primers in cDNA and gDNA. **(H and I)** Nuclear-cytoplasm separation RT-qPCR assay showed circNDRG1 was predominantly localized in cytoplasm. *P<0.05, **P<0.01 and ***P<0.001.

The differential expression of circRNAs between nitroxoline-treated and untreated bladder cancer cells were sequenced by circRNA microarray. Venn diagram and hierarchical clustering revealed that 31 circRNAs were co-expressed and up-regulated in J82, TCCSUP, T24 and UM-UC-3 cells (fold change ≥2) (Fig. 2A-B). In the current study, we focused on studying the function and molecular mechanisms of nitroxoline-mediated circRNAs in bladder cancer. After designing the specific primers for circRNAs, we screened out 6 circRNAs (hsa_circ_0085656, hsa_circ_0015296, hsa_circ_0039220, hsa_circ_0008590, hsa_circ_0060950, hsa_circ_0001593). Then the result of RT-qPCR assay performed that circNDRG1 (hsa_circ_0085656) was increased after the treatment of nitroxoline (10 µM) for 24h in 4 bladder cancer cell lines (Fig. 2D, Supplementary Fig. S1). Schematic illustration demonstrated circNDRG1 formation via exons 4-7 circularization of NDRG1 (Fig. 2C).

### Characterization of circNDRG1 in bladder cancer

To identify the circular structure of circNDRG1, we used RNase R to treat the total RNA of bladder cancer cell. The results showed that linear mRNA of NDRG1 was digested by RNase R, while circNDRG1 was resistant to RNase R for its unique circular structure (Fig. 2E). To test the stability of circNDRG1, total RNA was treated with transcription inhibitor Actinomycin D 19. The results performed that circNDRG1 had longer transcription half-life than linear mRNA of NDRG1 for its special circular structure (Fig. 2F). Furthermore, divergent primer was designed to amplify the circNDRG1 in cDNA and gDNA respectively, proving that circNDRG1 only existed in cDNA (Fig. 2G) 20. Nuclear-cytoplasm separation assay indicated that the distribution of circNDRG1 was more in the cytoplasm than in the nuclear (Fig. 2H-I).

### CircNDRG1 suppresses the metastasis of bladder cancer in vitro and vivo

**Figure 3:**
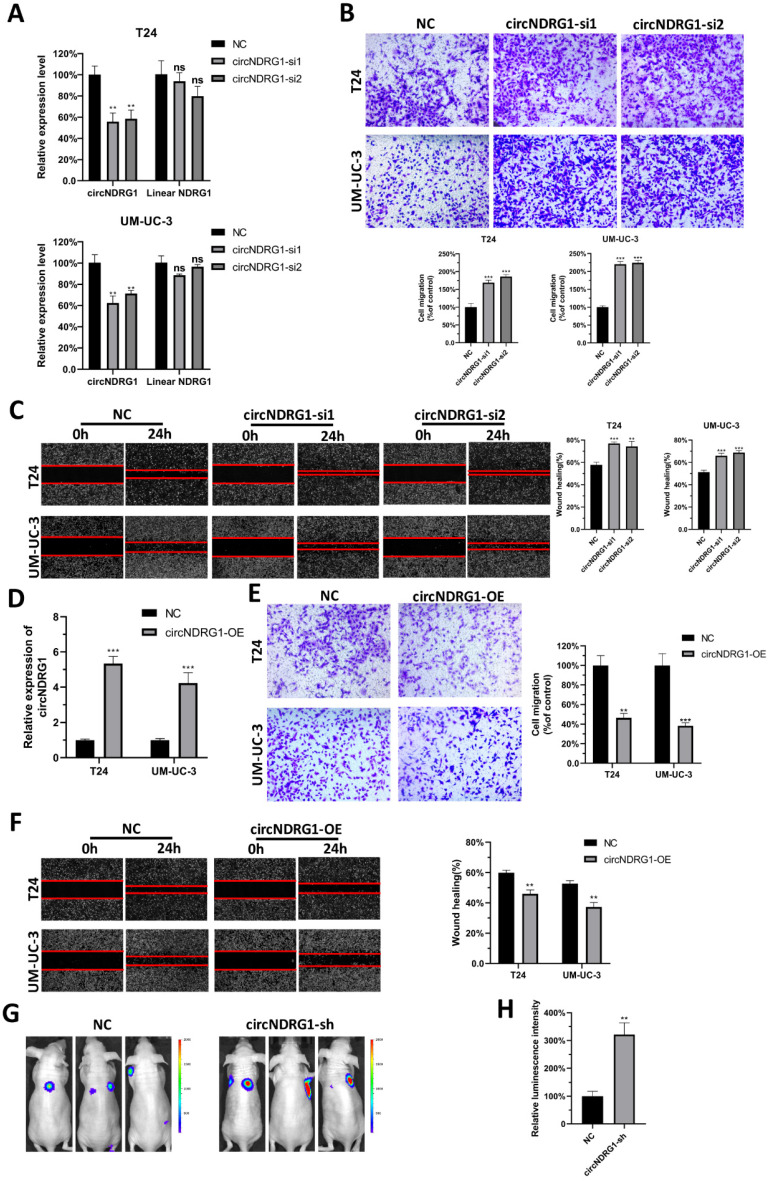
** CircNDRG1 inhibited the metastasis of bladder cancer in vitro and in vivo. (A)** The silence efficiency of circNDRG1 and linear mRNA NDRG1 by circNDRG1-si. **(B and C)** Transwell and wound-healing assay indicated the silence of circNDRG1 promoted the migration of bladder cancer cells. Cells were pretreated with nitroxoline (10 µM) for 24 hours. **(D)** The overexpression efficiency of circNDRG1 was determined by RT-qPCR. **(E and F)** Transwell and wound-healing assay showed the overexpression of circNDRG1 inhibited the migration of bladder cancer. Cells were pretreated with nitroxoline (10 µM) for 24 hours. **(G and H)** UM-UC-3 cells stably transfected with circNDRG1-sh and vector were injected into nude mice by tail veins and bioluminescent signals were measured by the IVIS imaging system. *P<0.05, **P<0.01 and ***P<0.001.

To investigate the biological roles of nitroxoline-mediated circNDRG1, we first designed 2 distinct siRNAs containing the back-spliced junction. The results displayed that siRNAs could silence the expression of circNDRG1 but did not influence the linear mRNA of host gene in T24 and UM-UC-3 cells (Fig. 3A). Then we transfected them into the cells treated with nitroxoline (10 µM) for 24 hours. Transwell assay and wound-healing assay results indicated that the knockdown of circNDRG1 promoted metastasis in bladder cancer cells significantly (Fig. 3B-C). Meanwhile, we designed an overexpression plasmid targeting circNDRG1, and stably transfected into T24 and UM-UC-3 cell respectively (Fig. 3D). The result demonstrated that circNDRG1 overexpression decreased the ability of metastasis (Fig. 3E-F). And transwell and wound-healing assay showed the consistent result with the treatment of nitroxoline (20 µM) for 24 hours (Supplementary Fig. S2). Furthermore, the effect on tumor metastasis was examined by using mouse tail-vein injection model. UM-UC-3 cells were infected with lentiviruses labelled with luciferase and intravenously injected into the mouse tail vein. Nitroxoline (30 mg/kg per mouse, fed for 2 days and rest for 1 day) by gavage was fed for 8 weeks in NC/circNDRG1-sh groups 13. The luminescence intensity of circNDRG1-sh group was stronger than the NC group significantly (Fig. 3G-H). Together, the results described above indicated that circNDRG1 suppressed the metastasis of bladder cancer in vitro and in vivo.

### EGR1 regulates the transcription of circNDRG1

**Figure 4:**
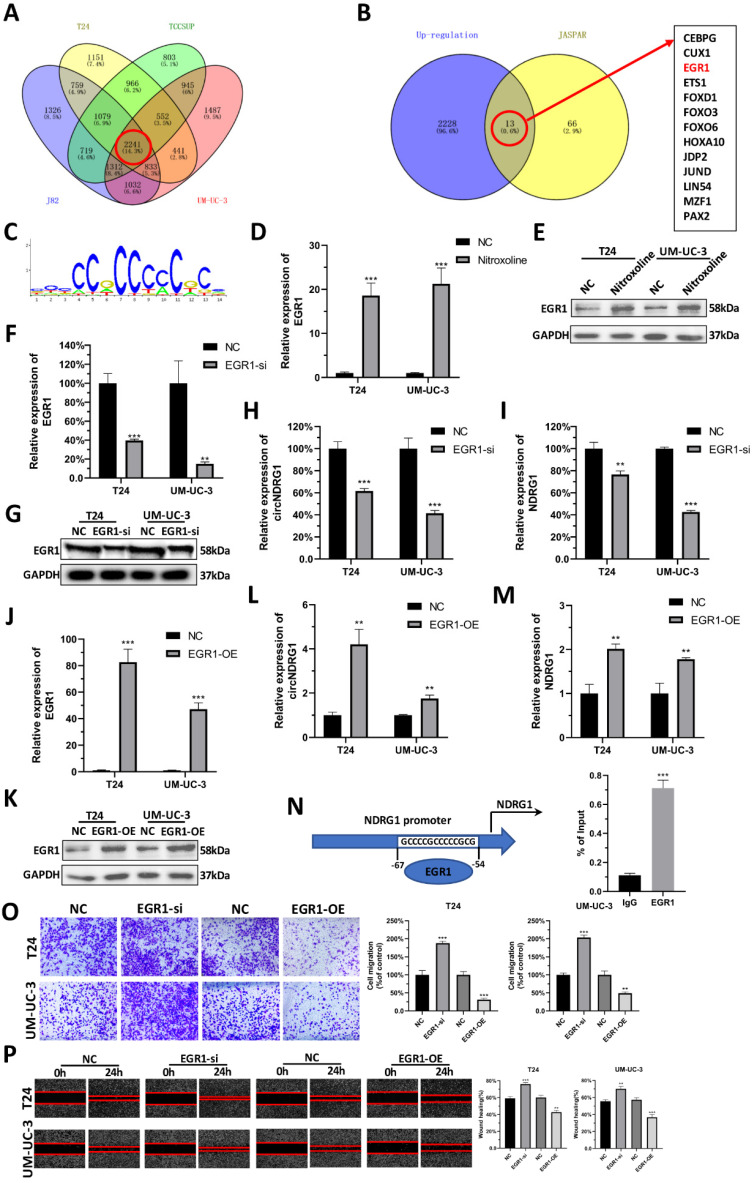
** Predicting EGR1 as upstream transcription factor of circNDRG1. (A and B)** Venn diagram representations of mRNA-seq in 4 bladder cancer cells and the intersection of predicted transcription factor from the JASPAR database. **(C)** The sequences binding with EGR1. **(D and E)** RT-qPCR and western-blotting assay to measure EGR1 mRNA and protein level after nitroxoline treatment. **(F and G)** EGR1 mRNA and protein measured by RT-qPCR and western-blotting assay after knocking down EGR1 by siRNA. **(H and I)** RT-qPCR showed the down-regulation of EGR1 contributed to the reduce of circNDRG1 and mRNA NDRG1. **(J and K)** EGR1-OE increased the mRNA and protein of EGR1 by RT-qPCR and western-blotting. **(L and M)** RT-qPCR showed overexpression of EGR1 activated circNDRG1 and mRNA NDRG1 expression. **(N)** ChIP assay of the binding of EGR1 to the promoter of host gene NDRG1. **(O and P)** Transwell and wound-healing assay showed that EGR1 inhibited the capacity of migration in bladder cancer. *P<0.05, **P<0.01 and ***P<0.001.

To explore the underlying mechanism of nitroxoline-mediated up-regulation of circNDRG1, we tested whether transcription factors can regulate the expression of circNDRG1. Accordingly, we sequenced the differential mRNA expression between nitroxoline-treated and untreated groups in bladder cancer cell lines. Among these, a total of 2241 genes were up-regulated (fold change ≥2). We predicted the transcription factors in JASPAR database, in which 66 transcription factors had the binding domain for binding to the promotor of host gene NDRG1 when the relative profile score threshold was set to 95%. After taking the intersection, only 13 candidate genes were suggested common target genes (Fig. 4A-C). We then reviewed the whole transcription factors in Pubmed, and found that EGR1 may be the target gene. RT-qPCR and western blotting assay revealed that the mRNA and protein expression level of EGR1 increased after nitroxoline (10 µM) treatment for 24 hours (Fig. 4D-E). To specifically deplete EGR1, we designed siRNA targeting EGR1, which reduced the expression of EGR1 in mRNA and protein expression (Fig. 4F-G). In addition, RT-qPCR assay showed that the expression of circNDRG1 and linear mRNA NDRG1 were down-regulated after the knock-down of EGR1 (Fig. 4H-I). In contrast, cells transfected with EGR1 overexpression plasmid showed the opposite expression profile of EGR1, circNDRG1 and linear mRNA NDRG1 (Fig. 4J-M). Furthermore, ChIP assay revealed EGR1 could directly bind to the target sequences of NDRG1 promotor regions (Fig. 4N). Finally, transwell and wound-healing assay indicated that EGR1 inhibited the capacity of migration in bladder cancer cells (Fig. 4O-P). Taking together, these results indicated that EGR1 regulated the transcription of circNDRG1 via binding the NDRG1 promotor.

### CircNDRG1 acts as a sponge for miR-520h in bladder cancer

**Figure 5:**
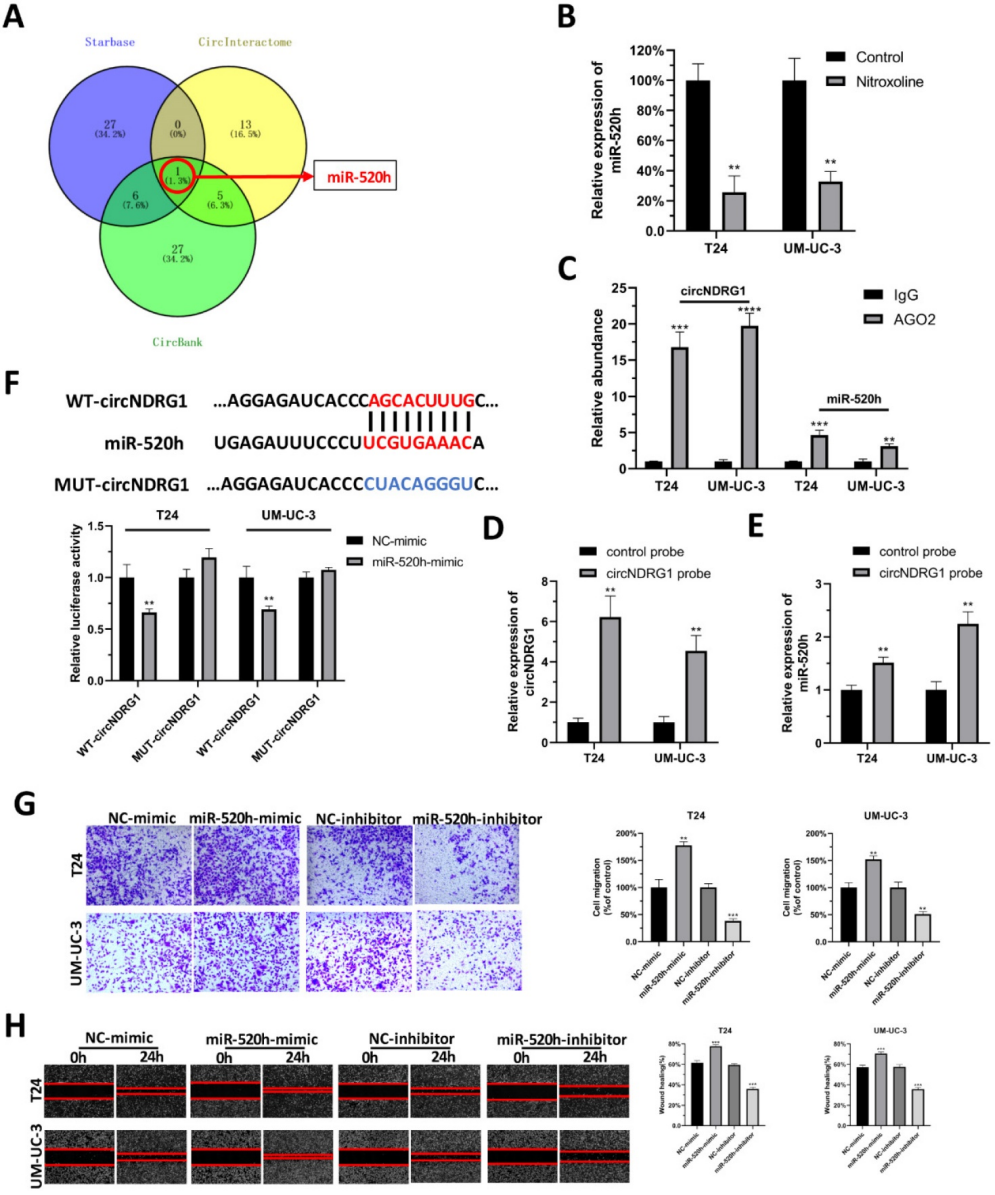
** CircNDRG1 acted as a sponge for miR-520h in bladder cancer. (A)** Venn diagram of the intersection among predicted microRNA by Starbase, CircInteractome and CircBank. **(B)** RT-qPCR showed nitroxoline suppressed the expression of miR-520h. **(C)** RIP experiments were carried out using an AGO2 antibody. **(D and E)** Biotin-labelled circNDRG1 probe was used to bind circNDRG1 through streptavidin agarose and miR-520h were sponged by circNDRG1. **(F)** Dual-luciferase reporter assay validated the binding between circNDRG1 and miR-520h. **(G and H)** Transwell and wound-healing assay showed miR-520h minic promoted the migration of bladder cancer cells, while miR-520h inhibitor inhibited the migration. *P<0.05, **P<0.01 and ***P<0.001.

It is generally accepted that circRNA could function as a molecular sponge for miRNA. Hence, we tried to explore the microRNA which might be sponged by circNDRG1. Three websites were chosen to predict the microRNA, including Starbase, CircInteractome and CircBank. After taking the intersection, we identified miR-520h as the only one predicted result (Fig. 5A). RT-qPCR assay confirmed that miR-520h decreased after the treatment of nitroxoline (10 µM) for 24h (Fig. 5B). RIP assay showed that circNDRG1 and miR-520h were highly enriched in anti-AGO2 group, indicating the interaction between circNDRG1 and miR-520h (Fig. 5C). RNA-pulldown assay indicated that circNDRG1 was able to directly pull down miR-520h, suggesting that circNDRG1 might directly and specifically sponge miR-520h (Fig. 5D-E). Dual luciferase assay further validated circNDRG1 functioned as a ceRNA for miR-520h in bladder cancer (Fig. 5F). In addition, transwell assay and wound-healing assay displayed miR-520h promoted the migration of bladder cancer cell lines (Fig. 5G-H).

### MiR-520h targets smad7/EMT

**Figure 6:**
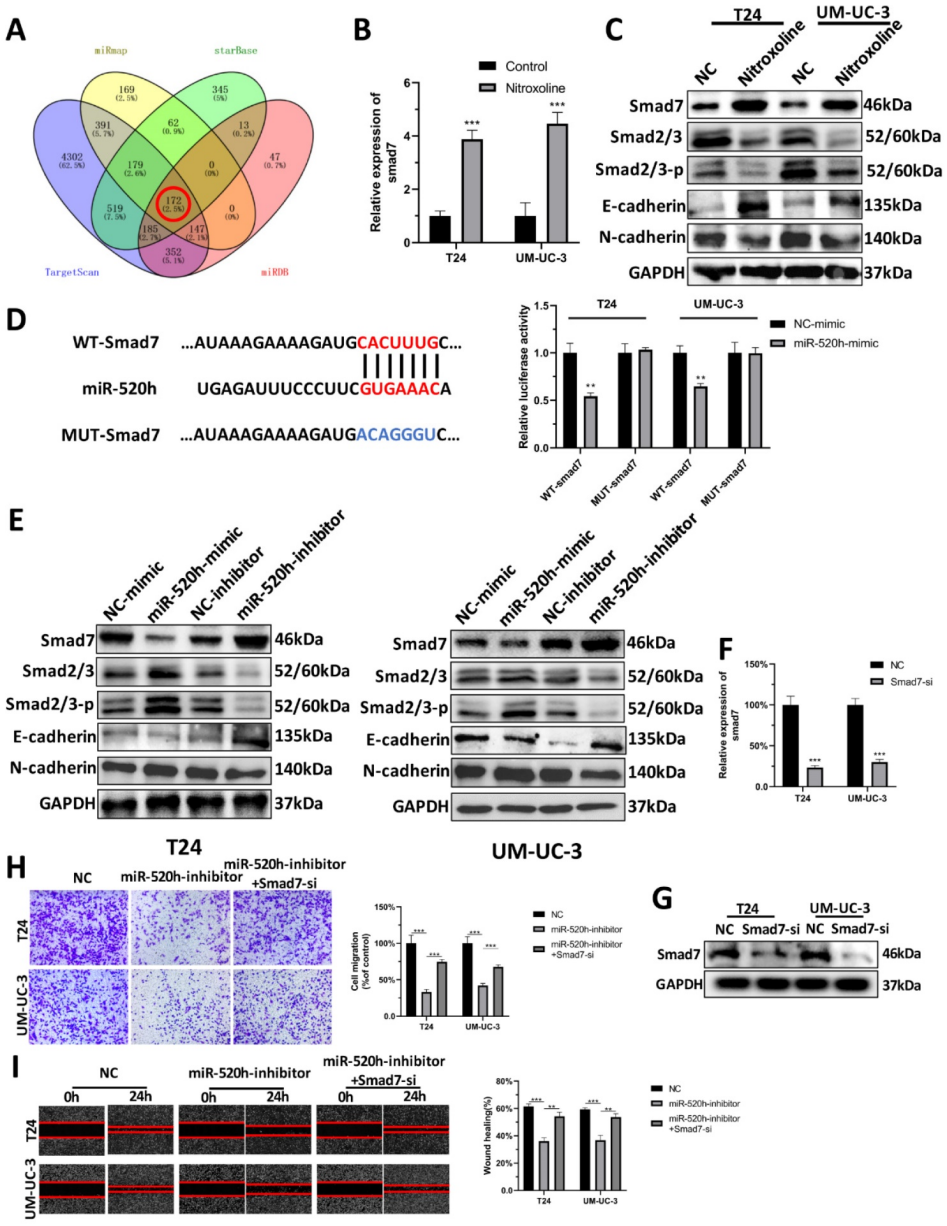
** Smad7 was the target of miR-520h and regulated the EMT. (A)** Venn diagram of the intersection among target genes by miRmap, Starbase, TargetScan and miRDB. **(B)** RT-qPCR assay demonstrated that nitroxoline led to increased smad7 expression. **(C)** Western-blotting assay demonstrated that nitroxoline up-regulated the expression of smad7, contributing to the repression of smad2/3 and EMT. **(D)** Dual luciferase assay showed the direct binding of miR-520h and 3'UTR of smad7 mRNA. **(E)** The exogenous dysregulation of miR-520h influence the expression of smad7, smad2/3 and EMT markers. **(F and G)** RT-qPCR and western blotting assay indicated the inhibition of smad7 after the transfection of smad7-si. **(H and I)** The capacity of migration was rescued by the decrease of smad7, which was inhibited by miR-520h-inhibitor. *P<0.05, **P<0.01 and ***P<0.001.

To screen the target gene of miR-520h, we overlapped the predicted results of miRmap, starBase, TargetScan and miRDB (Fig. 6A). The results for the remaining 172 genes analyzed can be seen in Supplementary (Table S2). Based on our previous findings, we found that nitroxoline inhibited the progression of bladder cancer via regulating EMT. Numerous reports in literature have identified the significant association between smad7 and EMT 21. We thus speculated that smad7 might be a potential target gene of miR-520h regulating the EMT signaling pathway. RT-qPCR assay and western-blotting assay demonstrated that nitroxoline (10 µM) for 24h resulted in increased smad7 level, leading to the repression of EMT (Fig. 6B-C). Dual luciferase assay showed that miR-520h targeted the 3'-UTR of smad7 mRNA to repress translation (Fig. 6D). Western blotting was used to evaluate the effect of miR-520h on the levels of smad7 and EMT markers. MiR-520h overexpression upregulated the expression of N-cadherin and smad2/3, and downregulated E-cadherin and smad7. In contrast, miR-520h inhibitor shows an opposite trend. Taken together, miR-520h was found to specifically bind smad7 and promoted the metastasis by targeting smad7/EMT in bladder cancer (Fig. 6E). Furthermore, we designed smad7-si to reduce the expression of smad7 in bladder cancer cells (Fig. 6F-G). And transwell and wound-healing assay showed that the inhibition of migration caused by miR-520h-inhibitor, could be rescued by silencing the expression of smad7 (Fig. 6H-I).

### Nitroxoline suppresses metastasis via circNDRG1/miR-520h/smad7/EMT pathway

**Figure 7:**
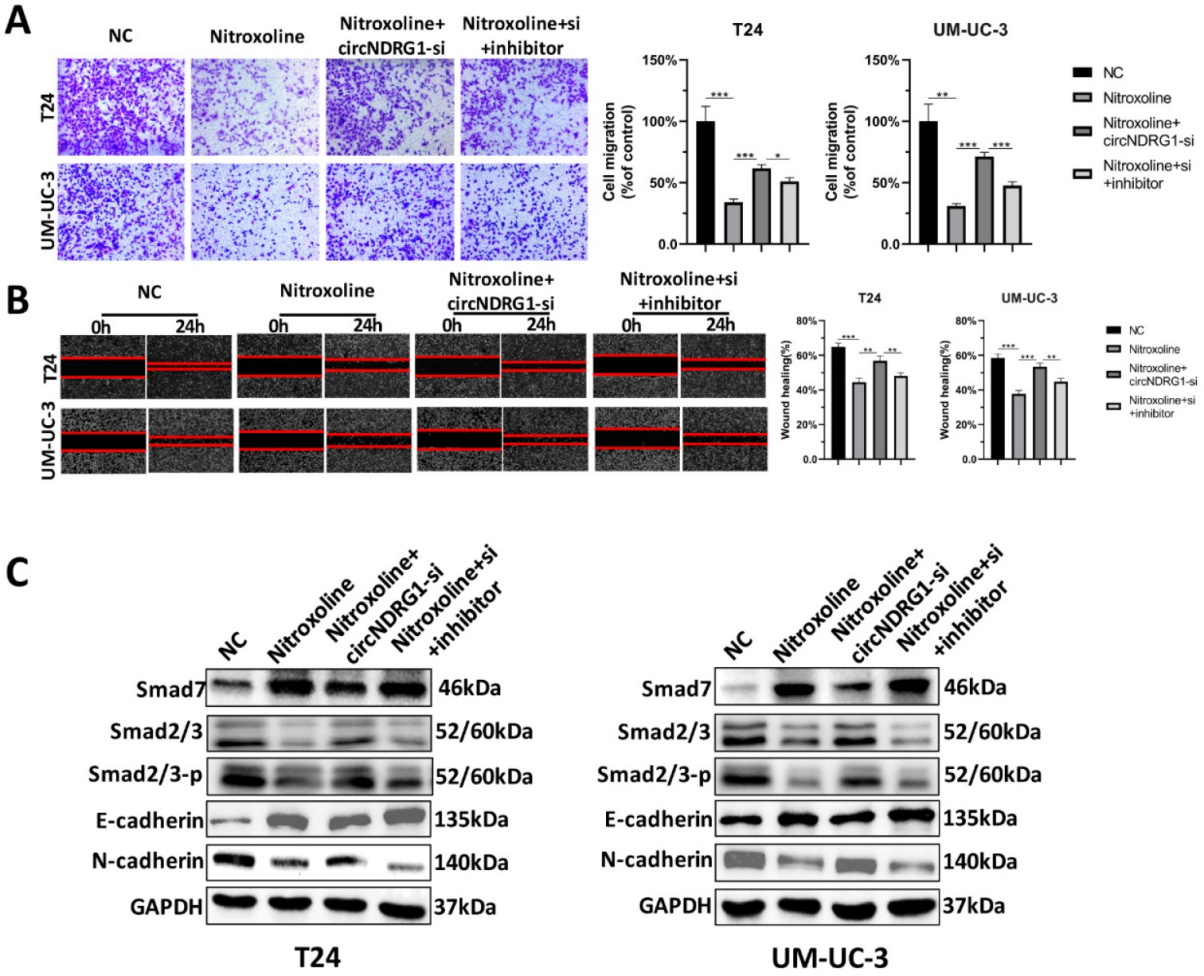
** CircNDRG1 regulated smad7 expression and inhibited metastasis by targeting miR-520h. (A and B)** Transwell and wound-healing assay demonstrated nitroxoline could inhibit the migration of bladder cancer cells, circNDRG1-si further promoted the migration, which was significantly rescued by preventing the expression of miR-520h. **(C)** Western-blotting assay showed that alteration of the expression levels of smad7, smad2/3 and the EMT-associated markers E-cadherin and N-cadherin, caused by nitroxoline plus circNDRG1-si treatment, were rescued by transfection with the miR-520h inhibitor. *P<0.05, **P<0.01 and ***P<0.001.

Rescue experiments were designed to identify the nitroxoline-mediated relationship of circNDRG1, miR-520h and smad7 in bladder cancer. Transwell assay and wound-healing assay showed that nitroxoline (10 µM for 24h) could inhibit the migration of bladder cancer cells, circNDRG1-si further promoted the migration, which was significantly rescued by preventing the expression of miR-520h (Fig. 7A and B). Western-blotting assay suggested that alteration of the expression levels of smad7, smad2/3, the EMT-associated markers E-cadherin and N-cadherin, caused by nitroxoline plus circNDRG1-si treatment, were rescued by transfection with the miR-520h inhibitor (Fig. 7C). In brief, these data revealed that nitroxoline suppressed metastasis via circNDRG1/miR-520h/smad7/EMT pathway and circNDRG1 positively regulated smad7/EMT by sponging miR-520h in bladder cancer.

## Discussion

Nitroxoline was a new potential antineoplastic drug, which had been confirmed in some cancer. Nitroxoline was first screened from the library of 175,000 compounds as Type 2 methionine aminopeptidase (MetAP2) protein inhibitor. MetAP2 was a potential antiangiogenic target and nitroxoline showed the anticancer activity in mouse models of human breast cancer and orthotopic bladder cancer xenografts 7, 22. Another research revealed that nitroxoline inhibited the tumor progression of glioblastoma cells by targeting caspase 3 and cleaved poly (ADP-ribose) polymerase 9. Other studies reported that nitroxoline exerted the antitumor effect by inducing Fork head box M1 (FoxM1) inhibition 23, or inhibiting bromodomain-containing proteins (BRDs) activity 24-27. Nitroxoline was also found to regulate the signaling pathway PTEN/Akt/AMPK/mTOR 28, 29, or p53 axle 8, 30. Furthermore, nitroxoline has been confirmed to be the inhibition of cathepsin B activity 31-35. Other studies have found EMT could be regulated by cathepsin B, leading to the metastasis of cancer 36, 37. And another research has reported nitroxoline suppressed the expression of β-catenin 38. Previous studies have confirmed that smad-complexes could regulate β-catenin, leading to the change of E-cadherin 21. For bladder cancer, our previous research also confirmed that nitroxoline reverses the process of EMT 13. The current research further verified that nitroxoline suppressed metastasis in bladder cancer through EMT pathway. The regulation of circNDRG1 to EMT might be another signaling pathway.

CircRNAs were special non-coding RNAs with circular structure. Many studies had reported the biological function of circRNAs in the progress of bladder cancer. CircPPP1CB suppressed tumorigenesis via miR-1703-3p/SMG1 axis 39; has_circ_modulated progression via miR-361-3p/IGF2R axis 40; circTAF4B promoted tumor progression through miR-1298-5p/TGFA axis 41. However, few researches tried to explore the relationship between antineoplastic drugs and circRNAs. In this study, we first uncovered that circNDRG1 functioned as the primary pharmacological biomarker responsible for nitroxoline's antitumor actions.

There are an increasing number of studies on the role of circRNAs in bladder cancer, but the regulation mechanism of the upstream transcription factor of circRNAs remains unclear 42. This research predicted several transcription factors regulating the expression of circNDRG1 by mRNA-microarray and bioinformatic analysis. The ChIP assay identified that EGR1 bound to the promotor of host gene and enhanced the expression of NDRG1. One study has reported that EGR1 could localize to the NDRG1 proximal promotor and regulate cell proliferation and survival in breast cancer 43. Furthermore, RNA-pulldown assay confirmed that miR-520h was sponged by circNDRG1, which was a common mechanism if circRNA was mostly in the cytoplasm.

MiR-520h, can exert both positive and negative effects on gene regulation in different cancers. Studies have reported that miR-520h induced pancreatic cancer cell death 44, while promoted tumor growth and progression in hepatocellular carcinoma cell 45, breast cancer 46 and epithelial ovarian cancer 47. Our study revealed that miR-520h inhibited the translation of smad7, which further may lead to the suppression of the EMT process. Here, we proposed a complete nitroxoline-mediated signaling pathway from EGR1 engagement to circNDRG1/miR-520h/smad7/EMT activation in bladder cancer. It was the first research to find the nitroxoline-mediated circRNA signaling pathway. However, we could not explain how nitroxoline regulate the expression of EGR1, and did not screen the direct target protein of nitroxoline so far to help explain its mode of action.

## Conclusion

We provided the evidence that circNDRG1 played an important role in nitroxoline-mediated suppression of the metastasis in bladder cancer. Our study proved that transcription factor EGR1 modulated the level of circNDRG1 by binding the promotor of NDRG1. And circNDRG1 sponged miR-520h contributing to the expression of smad7, which suppressed EMT phenotype. This founding indicated nitroxoline suppressed metastasis through the activation of EGR1/circNDRG1/miR-520h/smad7/EMT signaling pathway and circNDRG1 may become a potential biomarker during nitroxoline treatment for bladder cancer.

## Supplementary Material

Supplementary figures and table.

## Figures and Tables

**Figure 8:**
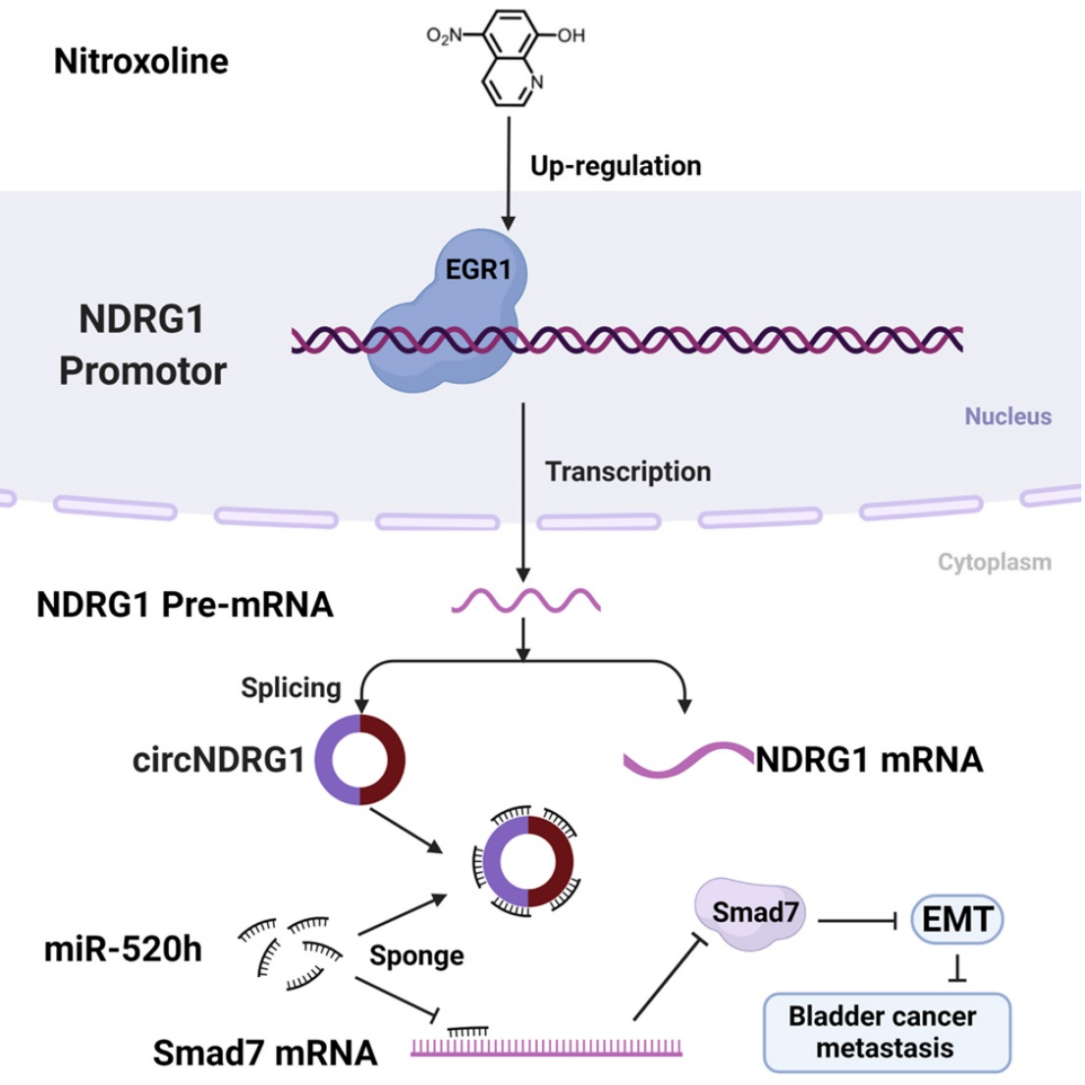
A schematic diagram depicting the mechanism of nitroxoline-mediated EGR1/circNDRG1/miR-520h/smad7/EMT signaling pathway in bladder cancer.

## Abbreviations

NMIBC: Non-muscle-invasive bladder cancer
MIBC: Muscle-invasive bladder cancer
BCG: Bacille Calmette-Guérin
EMT: Epithelial Mesenchymal Transition
CircRNA: Circular RNA
EGR1: Early Growth Response 1
Smad7: Smad family member 7
NDRG1: N-myc downstream regulated 1
ChIP: Chromatin immunoprecipitation
RIP: RNA immunoprecipitation
IVIS: In Vivo Image System
MetAP2: Type 2 methionine aminopeptidase
FoxM1: Fork head box M1
BRDs: Bromodomain-containing proteins
