# Supplementary material for: Nitroxoline suppresses metastasis in bladder cancer via EGR1/circNDRG1/miR-520h/smad7/EMT signaling pathway
Source: Int J Biol Sci. 2022 Aug 8;18(13):5207–20. doi: 10.7150/ijbs.69373 (PMC9379395; doi:10.7150/ijbs.69373)
Supplement: Supplementary file 1 — Supplementary figures and table. [file ijbsv18p5207s1.pdf]

Table S1. The sequence of primers, si-RNA and oligonucleotides

**Primer**	
circNDRG1 F	GGCTGAAATGCTTCCTGGAG
circNDRG1 R	TCTCGATGTCCTGCTCAGC
hsa_circ_0015296 F	TCGGCTTGACTACACTGTGT
hsa_circ_0015296 R	AGGCTTGCTCTTTAGGACCTT
hsa_circ_0039220 F	TCCCCATCCCACAATATCCC
hsa_circ_0039220 R	GCTAGCACTGTAGGACCCTG
hsa_circ_0008590 F	TGCTTCCAGGCCTCATATCG
hsa_circ_0008590 R	GGATGCCCAGGTTGTTAAAATCT
hsa_circ_0060950 F	CTTCCCAGCGCATTCCTTTG
hsa_circ_0060950 R	AGATCGGTCCATGATGCCAG
hsa_circ_0001593 F	GAAGCCCAGCCCGAGAAG
hsa_circ_0001593 R	GCTGCGGTGCTGGAGTAC
NDRG1 F	CCAACAAAGACCACTCTCCTC
NDRG1 R	CCATGCCCTGCACGAAGTA
GAPDH F	GTCAAGGCTGAGAACGGGAA
GAPDH R	AAATGAGCCCCAGCCTTCTC
EGR1 F	GGTCAGTGGCCTAGTGAGC
EGR1 R	GTGCCGCTGAGTAAATGGGA
U6 F	CTCGCTTCGGCAGCACA
U6 R	AACGCTTCACGAATTTGCGT
miR-520h rt	GTCGTATCCAGTGCAGGGTCCGAGGTATTCGCACTGGATACGACACTCTA
miR-520h F	CGCGACAAAGTGCTTCCCTT
miR-520h R	AGTGCAGGGTCCGAGGTATT
ChIP F	CCCCTACGACTGCTTGCG
ChIP R	GGGCGGAGGGCGACTTTAT
**Si-RNA**	
circNDRG1-si1	CGATTTGCTGAGCAGGACA
circNDRG1-si2	CTCGATTTGCTGAGCAGGA
hsa_circ_0039220-si1	CACAGGGTCCTACAGTGCT
hsa_circ_0039220-si3	CCACAGGGTCCTACAGTGC
EGR1-si	GGACAAGAAAGCAGACAAATT
Smad7-si	CCAAUGACCACGAGUUUAUTT
miR-520h minic	ACAAAGUGCUUCCCUUUAGAGU
miR-520h inhibitor	ACUCUAAAGGGAAGCACUUUGU
**Biotinylated probes**	
Biotin-circNDRG1	GGATTGAGCTAAACGACTCGTCCTGTAGCTCT

Table S2. The websites predicted target genes of miR-520h

ANXA4	ZNF200	GOLM1	SNRPD3	SPTY2D1	TBL1X
CAMK2N1	CYB5B	AKAP11	C16orf72	DGKE	SENP1
HIPK3	TTPAL	KLHL15	PPP2R3A	IFNAR1	CELSR3
TBC1D15	RGPD5	SDC2	RAB39B	SS18L1	CBX5
UGP2	RAP2C	MAN1A1	SMAD7	SCML2	ANKRD50
RRAGD	GMCL1	LCLAT1	G3BP1	ITGAV	RAP2B
RTCA	TMEM123	KPNA1	SLC31A1	DCBLD2	MXD1
PBK	G2E3	GPR137C	VAV3	MSL2	RACGAP1
FBXL3	GPCPD1	ZFP91	ELK4	AHCTF1	KCTD20
PRCP	VLDLR	TAF5L	SPTLC2	TNRC6B	ZNF543
SERINC1	SPTSSA	SLC45A4	INPP5F	EFNB2	ACBD5
PFN2	ANLN	ABL2	LARP4B	OCRL	BTBD7
MSANTD4	PIAS2	BRD1	XPO4	SLC5A3	MGAT4A
PEX11B	INSIG2	RAB22A	TMEM245	SAMD8	COL5A2
SNW1	FCF1	LIN7C	ETF1	ATAD2B	NBEA
F3	JAZF1	GNS	CEP57	SPRY4	SMAD6
SLC35A5	CPSF6	FXR1	ATXN1	SLC39A10	NUFIP2
LUC7L3	GCH1	ZNF451	SSR1	TGFBR1	KAT6A
CLOCK	CEP128	PLOD2	PDE4A	KIF11	RICTOR
VPS26A	RAB11B	TNRC6A	HIF1A	CCNB1	AZIN1
RND3	UBE2W	ABHD2	PPP6C	KPNA3	LATS1
IRX3	S1PR3	SATB2	MEF2A	GXYLT1	WNK1
PRKAB2	PITRM1	MBTPS2	LIN54	PRICKLE2	
CREBRF	DBN1	LIN28B	TMEM64	ARAP2	
GNAI3	PRRG4	RAB21	HIPK2	EDEM1	
PTK2	HMGCS1	THRAP3	ZBTB6	PIK3R1	
MMP2	EIF2S1	KIAA1958	OTUD4	RIT1	
SEC23IP	ZNF618	ZNF697	SLK	PRLR	
PLEKHA1	BCOR	ANKLE2	INO80D	MID1	
RGPD6	SMAD3	CAPN7	CHD2	PPARGC1B	

A

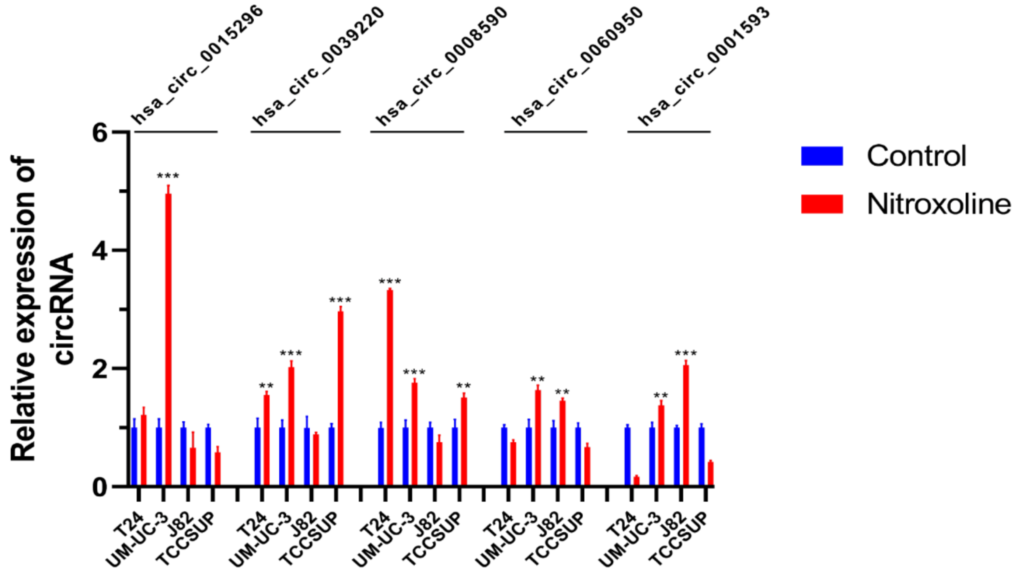

Fig. S1. The screening process of circRNAs. (A). RT-qPCR assay showed the change of expression of 5 circRNAs after the treatment of nitroxoline. *P<0.05, **P<0.01 and ***P<0.001.

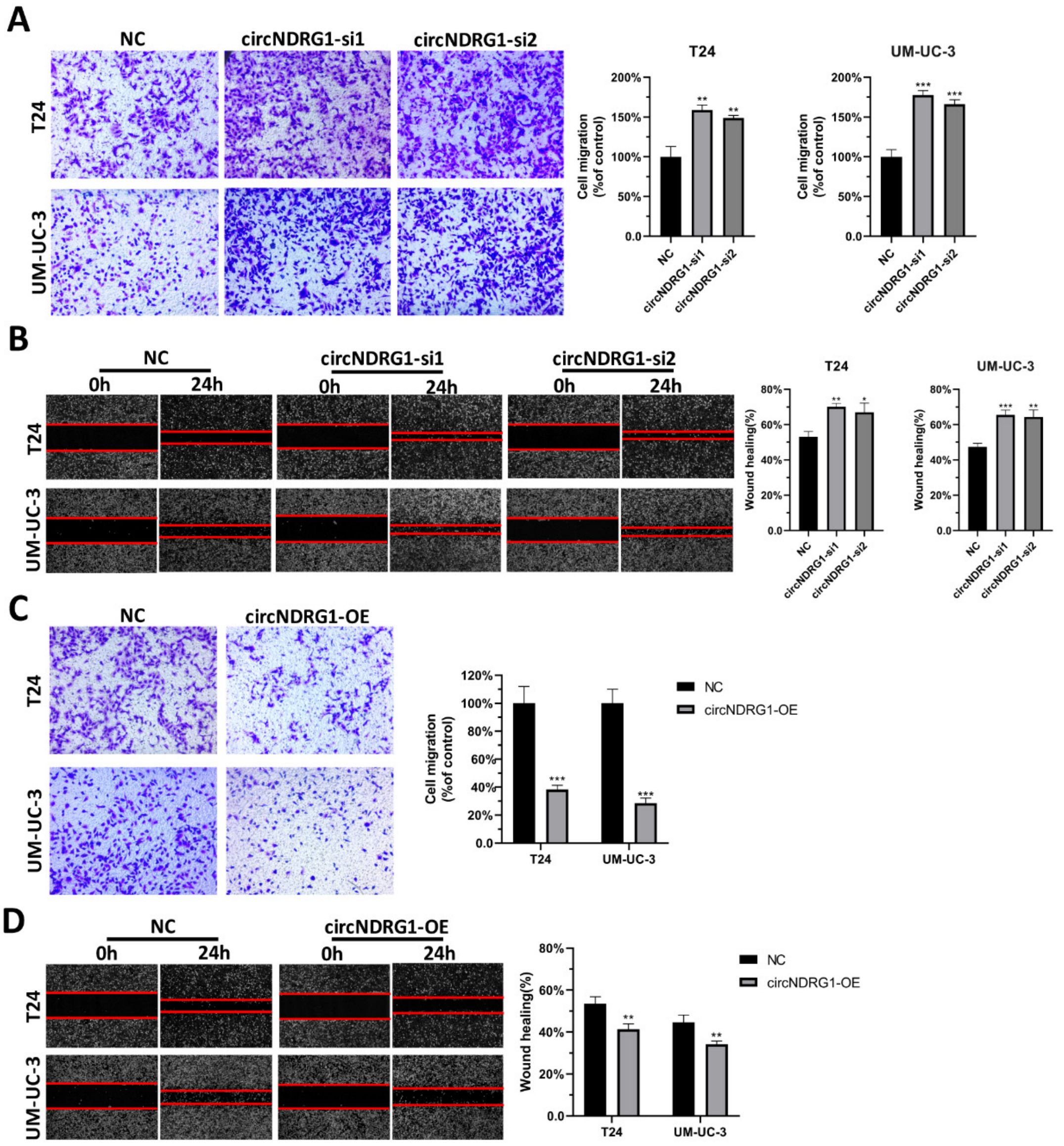

Fig. S2. CircNDRG1 inhibited the migration of bladder cancer after the treatment of nitroxoline (20 μM). (A and B). Transwell and wound-healing assay indicated the silence of circNDRG1 promoted the migration of bladder cancer cells. (C and D). Transwell and wound-healing assay showed the overexpression of circNDRG1 inhibited the migration of bladder cancer cells. Cells were pretreated with nitroxoline (20 μM) for 24 hours. **P*<0.05, ***P*<0.01 and ****P*<0.001.
